# Soil Environmental Conditions and Microbial Build-Up Mediate the Effect of Plant Diversity on Soil Nitrifying and Denitrifying Enzyme Activities in Temperate Grasslands

**DOI:** 10.1371/journal.pone.0061069

**Published:** 2013-04-17

**Authors:** Xavier Le Roux, Bernhard Schmid, Franck Poly, Romain L. Barnard, Pascal A. Niklaus, Nadine Guillaumaud, Maike Habekost, Yvonne Oelmann, Laurent Philippot, Joana Falcao Salles, Michael Schloter, Sibylle Steinbeiss, Alexandra Weigelt

**Affiliations:** 1 Université de Lyon, INRA, CNRS, Université Lyon 1, Microbial Ecology Centre (UMR 5557 CNRS, USC 1364 INRA), Villeurbanne, France; 2 Institute of Evolutionary Biology and Environmental Studies, University of Zurich, Zurich, Switzerland; 3 Institute of Plant Sciences, ETH Zurich, Zurich, Switzerland; 4 Max Planck Institute for Biogeochemistry, Jena, Germany; 5 Geoecology, University of Tübingen, Tübingen, Germany; 6 Laboratoire de Microbiologie des Sols, INRA, Dijon, France; 7 Research Unit for Environmental Genomics, Helmholtz Zentrum München, Neuherberg, Germany; 8 Institute of Ecology, Friedrich Schiller University, Jena, Germany; Dowling College, United States of America

## Abstract

Random reductions in plant diversity can affect ecosystem functioning, but it is still unclear which components of plant diversity (species number – namely richness, presence of particular plant functional groups, or particular combinations of these) and associated biotic and abiotic drivers explain the observed relationships, particularly for soil processes. We assembled grassland communities including 1 to 16 plant species with a factorial separation of the effects of richness and functional group composition to analyze how plant diversity components influence soil nitrifying and denitrifying enzyme activities (NEA and DEA, respectively), the abundance of nitrifiers (bacterial and archaeal *amoA* gene number) and denitrifiers (*nirK, nirS* and *nosZ* gene number), and key soil environmental conditions. Plant diversity effects were largely due to differences in functional group composition between communities of identical richness (number of sown species), though richness also had an effect per se. NEA was positively related to the percentage of legumes in terms of sown species number, the additional effect of richness at any given legume percentage being negative. DEA was higher in plots with legumes, decreased with increasing percentage of grasses, and increased with richness. No correlation was observed between DEA and denitrifier abundance. NEA increased with the abundance of ammonia oxidizing bacteria. The effect of richness on NEA was entirely due to the build-up of nitrifying organisms, while legume effect was partly linked to modified ammonium availability and nitrifier abundance. Richness effect on DEA was entirely due to changes in soil moisture, while the effects of legumes and grasses were partly due to modified nitrate availability, which influenced the specific activity of denitrifiers. These results suggest that plant diversity-induced changes in microbial specific activity are important for facultative activities such as denitrification, whereas changes in microbial abundance play a major role for non-facultative activities such as nitrification.

## Introduction

In a context of unprecedented rates of species extinction and human-induced shifts in the composition of biological communities, understanding how changes in plant diversity impact ecosystem functioning is of paramount importance [1], [2]. During the last decades, several studies have provided evidence of the functional importance of biodiversity for ecosystem processes [3], [4], [5], but the specific mechanisms underlying the observed patterns have remained unclear. The question for plant diversity–ecosystem functioning research is thus no longer whether plant diversity matters, but how it matters [6], [7]. More detailed analyses have shown that among the different components of biodiversity, the number of species (species richness) and functional groups (functional group richness), the presence and abundance of particular species or functional groups, and the diversity of species functional traits are the main drivers of plant diversity effects on ecosystem functioning [7], [8], [9].

However, many biodiversity studies to date have focused on the effect of biodiversity on primary productivity, while key soil processes and associated microbial activities have less been studied, despite their importance for ecosystem functioning and plant growth, but see [10], [11], [12], [13], [14], [15]. In the present study, we focus on enzyme activities involved in nitrification and denitrification. These two key steps of the soil nitrogen (N) cycle exert key controls on ecosystems from local to global scale. In particular, nitrification and denitrification control (i) soil inorganic N availability and subsequent primary production, and (ii) N losses from ecosystems. The latter includes nitrate leaching, which plays a crucial role in groundwater quality, and emissions of nitrogen monoxide and nitrous oxide that impact atmospheric chemistry and global warming [16], [17]. Biodiversity effects on these processes are therefore not only important for the ecosystem’s N budget and biomass production, but also for the way in which the ecosystem interacts with adjacent systems and the global climate [18]. Soil microorganisms are subject to specific ecological limitations and therefore may not respond like plant production to different components of biodiversity.

Soil nitrate is primarily produced by microbial oxidation of ammonium under aerobic conditions by autotrophic microorganisms during nitrification [19]. During the anaerobic process of denitrification [20], heterotrophic microorganisms utilize nitrate to oxidize available organic carbon compounds, releasing gaseous nitrous oxides and dinitrogen. Both processes respond sensitively to substrate availability and redox conditions. By altering the main driving variables of nitrification and denitrification (i.e. soil inorganic N availability, organic C availability, water content and oxygen availability, and pH), plant biodiversity can affect nitrification and denitrification. Several lines of evidence suggest that this is likely to take place:

1. Soil nitrate concentration has been shown to decrease with increasing plant diversity [21], [22]. Moreover, nitrifying activity in grassland was reduced with increasing plant diversity, likely due to enhanced capture of ammonium by plants [21], though other studies found no effect of plant diversity on nitrification [23], [24]. It is also well established that the presence of legumes generally increases N availability to plant communities [25], thereby largely governing N cycling and ecosystem functioning in many grassland biodiversity studies [1], [22].
2. The level or vertical distribution of soil water can be affected by plant diversity [26] which may subsequently affect nitrification or denitrification, though soil water content can be unaffected by plant diversity in temperate grasslands [27].
3. Dissolved organic carbon (DOC) provides a readily available C source to denitrifying soil microorganisms. While the factors regulating DOC concentrations in soils are not yet well understood, the documented plant diversity effects on primary production and rooting patterns could possibly also affect DOC levels, and thereby the activity of denitrifiers.
4. Plants produce compounds such as root exudates that affect microorganisms. In addition, nitrification and denitrification are sensitive to soil pH, and plants can acidify the rhizosphere and the surrounding soil. All these processes could vary with plant identity, which might affect nitrification and denitrification [28], [29].

Only a few studies have analysed the effect of plant diversity on potential nitrification and denitrification or the characteristics of nitrifying and denitrifying communities to date [11], [14], [15], [24], generally in mesocosms and on a short term (a few months). Moreover, these studies did not unravel the effects of plant species richness per se, species identity, and functional group presence/abundance, which requires to use a large pool of plant species, and did not hierarchize the importance of environmental factors and microbial abundances to explain the observed changes in potential microbial activities through causal relationships. Here we present a detailed analysis of plant diversity effects on nitrification and denitrification in a large-scale field trial (Jena Experiment) in which plant species richness and functional group presence/abundance were manipulated independently. All measurements were made in the 5^th^ and 6^th^ years after establishment of experimental plant communities, well clear of initial soil disturbance. The objectives of the study were (1) to identify the main components of plant diversity (richness vs. abundance or presence of key functional groups) that influence soil nitrifying and denitrifying enzyme activities, which are key characteristics influencing soil N dynamics; and (2) to assess the effects of plant diversity on nitrifying and denitrifying enzyme activities in relation to effects on putative environmental drivers of enzyme activities (soil moisture, C availability, nitrate and ammonium availability) and on abundances of nitrifiers and denitrifiers.

## Materials and Methods

### Ethics Statement

No specific permits were required for the described field studies. The Jena field site is a former arable land owned by an agricultural collective. The ground is leased by the Research consortium of the Jena experiment for scientific purpose including soil sampling and other experimental manipulations. The site is not protected in any way. The areas studied do not involve any species endangered or protected in Germany.

### Study System and Experimental Design

The present study was carried out as part of a large biodiversity experiment located in Jena, Germany (50°57′N, 11°37′W, elevation 130 m a.s.l.), [30]. The experimental study system was derived from a typical Central European mesophilic grassland community as it was traditionally used for haymaking. Sixty common plant species of this community were used and divided by ordination using 17 species traits into four functional groups that could be identified *a posteriori* as grasses, legumes, “tall” non-leguminous herbs, and “small” non-leguminous herbs, [30].

The Jena Experiment was sown in 2002 and used an almost fully orthogonal species richness x functional group composition design where species of all functional groups occurred in monoculture and single functional groups could make up communities of up to 16 species (Figure S1), [30]. The experimental area was partitioned into four blocks, containing a total number of 88 large plots (20×20 m). Experimental communities of 1, 2, 4, 8 and 16 species were established in the plots. Plant species were chosen from a pool of 60 species with the constraint that all plant functional groups were evenly represented at each level of plant species richness. Functional group composition is defined as the combination of functional groups represented in a community. Functional group composition could be decomposed into the following contrasts: factors for the presence/absence of each functional group, covariables for the proportions of each functional group if present, and interactions. An alternative decomposition of functional group composition into contrasts was a full factorial with the four factors of presence/absence of each functional group and all interactions of these factors. Because only 16 of the 60 species were established as monocultures on the large plots, the species that were not established in the large plots were grown on 44 additional 3.5×3.5 m plots. Previous work using the same plots has shown that plot size is unimportant for biodiversity–ecosystem functioning relationships [31]. In all mixtures, plant species were sown at maximum evenness. All plots were manually weeded regularly, thus maintaining plant species richness at the planned levels or slightly below in cases where a plant species did not establish or died. The strong correlation between the number of sown species and the number of observed target species (R^2^>0.99) verified the successful establishment of the species richness gradient. The experimental plant communities were mown twice per year and were not fertilized.

### Soil Sampling

Soil samples (0–8 cm depth) were collected using corers (8 cm diameter) in October 2006 at the end of the growing season. Ten samples were randomly taken for each large plot (total of 880 soil cores), and five samples were randomly taken for each small plot (additional 220 soil cores). For each plot, a composite soil core was obtained by pooling one half of each core taken in the plot. The remaining half-cores were kept as individual soil samples. For each composite or individual sample, fresh soil was sieved using 2-mm mesh size, homogenized and stored at +4°C a few days before measurement. A sub-sample was immediately frozen at −20°C before DNA extraction. Soils were also similarly sampled from all the large plots during another field campaign in October 2007.

### Denitrifying and Nitrifying Enzyme Activity Assays

Denitrifying enzyme activity, DEA, was measured in fresh soils according to Patra et al. [32] on all the composite soil samples in 2006 and 2007 and on individual soil samples (10 per plot) for 28 plots in 2006. DEA was determined as the linear rate of production of N_2_O during a 8-hour incubation (28°C, 200 µg NO_3_^–^N g^−1^ and 1 mg C g^−1^ added) using a gas chromatograph (Agilent P200, Santa Clara, CA, USA). A 90∶10 He-C_2_H_2_ atmosphere provided anaerobic conditions and inhibited N_2_O-reductase activity. Nitrifying enzyme activity, NEA, was measured on fresh soil samples as detailed by Patra et al. [32]. Soil nitrate content was measured before and after a 7-hour aerobic incubation (28°C, 200 µgN-(NH_4_)_2_SO_4_ g^−1^ added).

#### Quantification of nitrifier and denitrifier abundances

In early 2008, DNA was extracted for each frozen soil sub-sample of the October 2006 field campaign using the PowerSoil™ DNA Isolation Kit (MO BIO Laboratories, Carlsbad, CA, USA), and the abundance of denitrifiers was estimated by quantitative PCR (qPCR) targeting the genes encoding the catalytic subunit of the key enzymes of the denitrification pathway. Fragments of the *nirK*, *nirS* and *nosZ* genes encoding the copper and *cd*_1_ nitrite reductases and the nitrous oxide reductase, respectively, were amplified as described by Baudoin et al. [33]. All assays were run using known copy number of linear plasmids containing targeted genes from either *Bradyrhizobium japonicum* USDA110, *Pseudomonas aeruginosa* PAO1, *Agrobacterium tumefaciens* C58 or *Sinorhizobium meliloti* 1021, containing known copy numbers of targeted genes as external standards [34].

Though nitrification is a two step-process, we targeted the first one, ammonia oxidation, as it is often assumed to limit nitrification rate [19]. The abundance of *amoA* gene copies from ammonia oxidizing bacteria (AOB) was quantified using a SybrGreenI-based real-time PCR technique as described in Leininger et al. [35] with the exception that the cloned *amoA* gene of *Nitrosomonas multiformis* ATCC25196 was used as standard. The abundance of *amoA* gene copies from ammonia oxidizing archeae (AOA) was measured by quantitative PCR targeting the archaeal *amoA* gene, according to the methodology described in Le Roux et al. [36]. Melting curve analysis confirmed the specificity of the amplification, and amplification efficiencies higher than 80% were obtained for all PCR reactions. All measurements were performed in triplicate.

For all quantitative PCR approaches, possible inhibitory effects of co-extracted humic compounds in soil extracts were checked by dilution series, but no inhibition was observed.

### Soil Environmental Variables

Soil moisture was determined by the gravimetric technique for the samples collected in 2006. Soil ammonium and nitrate concentrations were determined by extracting a fresh soil sub-sample equivalent of 5 g dry soil with 50 mL of 2 M KCl for 30 min. After filtration, the extracts were analysed for ammonium and nitrate by continuous flow analysis (Skalar, Breda, Germany) according to Oelmann et al. [37]. Dissolved organic C (DOC) in soil solution was determined using an elemental analyzer (high-TOC, Elementar Analysensysteme GmbH, Hanau, Germany) in soil solution collected during a 2-week period around sampling dates (glass suction plates with 1 µm pore size installed at 10 cm depth; UMS GmbH, Munich, Germany). Soil pH was not affected by treatments so that pH was not included as a possible driver of observed changes in soil enzyme activities.

### Statistical Analyses

The enzyme activity data were analyzed by multiple regression [38]. First, spatial variation was eliminated as a block effect and with covariables X and Y for north–south and west–east trends. We fitted a two-dimensional response surface of the type X+Y+X*Y+X^2^+Y^2^+X^2^*Y^2^ after the block term and then dropped all terms that were not significant from any particular model. After accounting for spatial variation by fitting these terms (which are summarized in the ANOVA tables 1, 2 and S2 in a single line), the treatment factors species richness, decomposed into a log-linear contrast (logSR) and deviation (SR), and the presence and initial sowing proportion of each of the four functional groups were fitted. Terms that were not significant were dropped from the model, resulting in the simplified models shown in tables 1 and S1 (the SR term was kept in the model for DEA in 2007, even though it was not significant, to help comparison with results obtained for 2006 activity data). Replacing initial sowing proportions of functional groups with actual aboveground biomass proportions yielded very similar results. To investigate the mechanisms explaining the observed plant diversity effects, we (i) added environmental soil covariables (soil moisture, soil nitrate, soil ammonium, and DOC) and microbial abundance data to the regression models, fitting them after the terms for spatial variation but before the plant diversity terms, and (ii) used path analysis to test potential causal relationships between plant diversity terms, environmental covariables, microbial abundance, and soil enzyme activities. The path analyses were calculated with variance-covariance matrices to assess significances of path coefficients; the standardized solutions (corresponding to the analysis of correlation matrices) are presented here.

**Table 1:** ANOVA results of the multiple regression models fitted for the two dependent variables nitrifying and denitrifying enzyme activities for October 2006, using plant diversity components only as explanatory variables.

Nitrifying enzyme activity				
Source of variation	d.f.	s.s.	m.s.	F pr.
Spatial variation	4	1.237	0.309	<.001
LogSR	1	0.183	0.183	0.009
Legume abundance (% sown)	1	2.446	2.446	<.001
Residual	69	1.755	0.025	
Total	75	5.62	0.0749	
**Denitrifying enzyme activity**				
**Source of variation**	**d.f.**	**s.s.**	**m.s.**	**F pr.**
Spatial variation	6	14.29	2.38	<.001
logSR	1	0.96	0.96	0.004
SR	3	1.05	0.35	0.025
Presence of legumes	1	2.57	2.57	<.001
Grass abundance (% sown)	1	0.54	0.54	0.027
Residual	63	6.62	0.11	
Total	75	26.03	0.35	

LogSR and SR respectively refer to the decomposition of the species richness factor into a contrast for log-linear richness and remainder (deviation from log-linearity). For each table, values are presented only for variables that had a significant effect on activity. ANOVA results for 2007 are very similar and are presented in Table S1. d.f.: degree of freedom; s.s.: sum of squares; m.s.: mean sum of squares; F pr.: significance level (F-test).

**Table 2:** ANOVA results of the multiple regression models fitted for the two dependent variables NEA and DEA for October 2006, using soil variables, microbial abundances and plant diversity components as explanatory variables.

Nitrifying enzyme activity				
Source of variation	d.f.	s.s.	m.s.	F pr.
Spatial variation	4	1.24	0.31	<.001
Ammonium	1	0.16	0.16	0.009
AOB abundance	1	0.65	0.65	<.001
logSR	1	0.02	0.02	NS
Legume abundance (% sown)	1	2.03	2.03	<.001
Residual	66	1.51	0.02	
Total	74	5.61	0.08	
**Denitrifying enzyme activity**				
**Source of variation**	**d.f.**	**s.s.**	**m.s.**	**F pr.**
Spatial variation	6	14.29	2.38	<.001
Moisture	1	1.51	1.51	<.001
Nitrate	1	2.27	2.27	<.001
*nirK* number*	1	0.25	0.25	NS
logSR	1	0.22	0.22	NS
Presence of legumes	1	0.97	0.97	0.002
Grass abundance (% sown)	1	0.47	0.47	0.03
Residual	62	5.98	0.10	
Total	74	25.95	0.35	

* results remain unchanged when considering nirS- or nosZ-harbouring bacteria.

LogSR and SR respectively refer to the decomposition of the species richness factor into a contrast for log-linear richness and remainder (deviation from log-linearity). For each table, values are presented only for variables that had a significant effect on activity. In particular, dissolved organic carbon explained only little variation and was therefore not included. Abbreviations are as in Table 1.

Although technical replicates that went into each single data point were sometimes used (in particular for microbial abundance data), we never used technical replicates to compute error terms in the analysis but always averaged them for each biological replicate. Thus, the residual means squares in Tables 1 and 2 give the among-plot residual variation which is the appropriate error for our statistical tests, and in path analyses errors can be seen by the arrows on the dependent variables with no causal variable at the beginning of the arrow.

## Results

### Effects of Components of Plant Diversity on NEA and DEA

**Figure 1:**
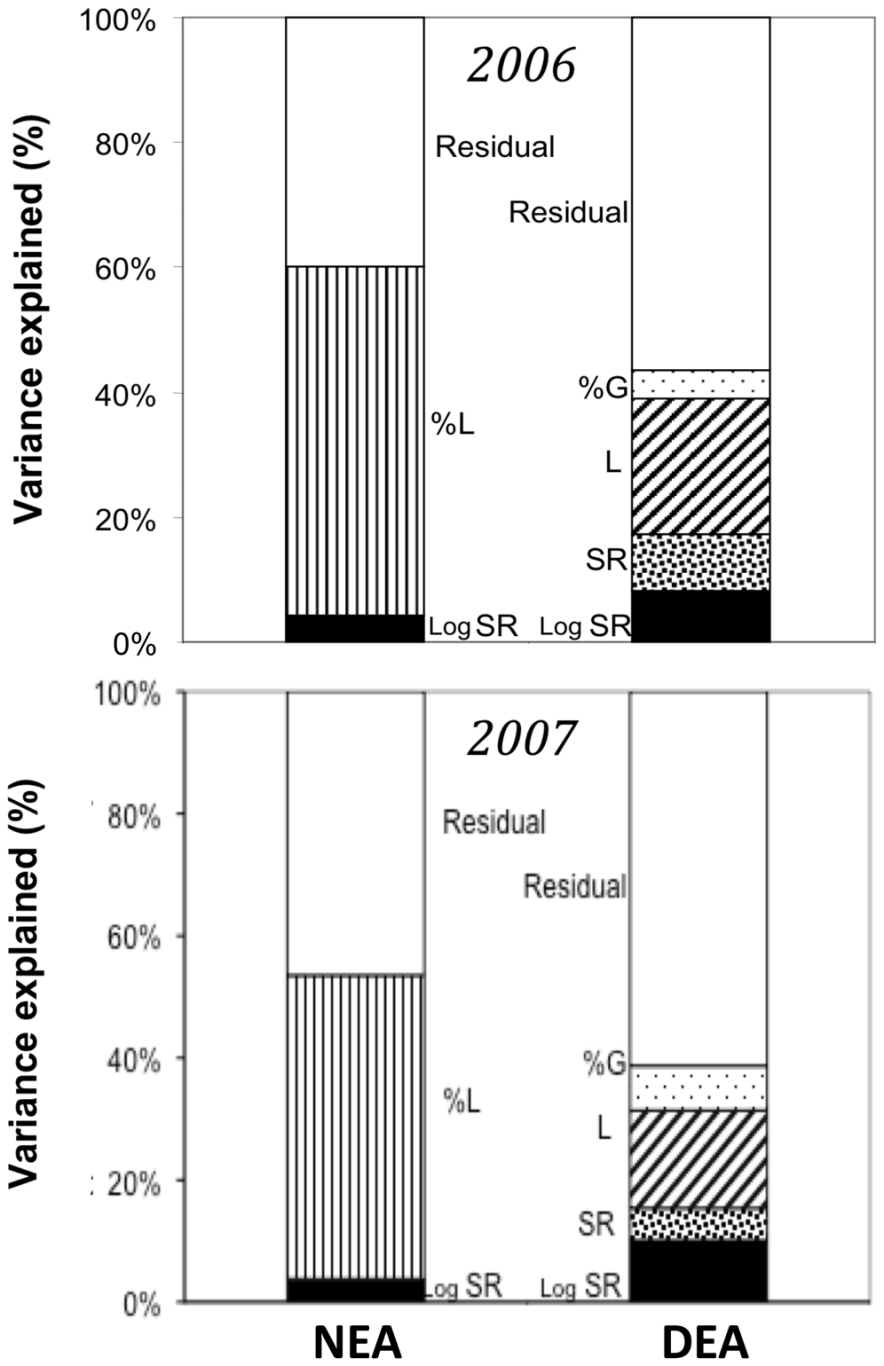
Plant Diversity Influence On Soil Enzyme Activities. Percentages of variation in nitrifying and denitrifying enzyme activities, respectively NEA and DEA, significantly explained by components of plant diversity. Data are presented (top) for 2006, and (bottom) for 2007. LogSR and SR refer to the decomposition of the species richness factor into a contrast for log-linear richness and deviation from log-linearity, respectively. L refers to presence of legumes, and %L and %G refer to the percentage of legumes and grasses, respectively. Significance levels of the effects of plant diversity components observed in 2006 and 2007 are presented in Table 1 and Table S1, respectively.

**Figure 2:**
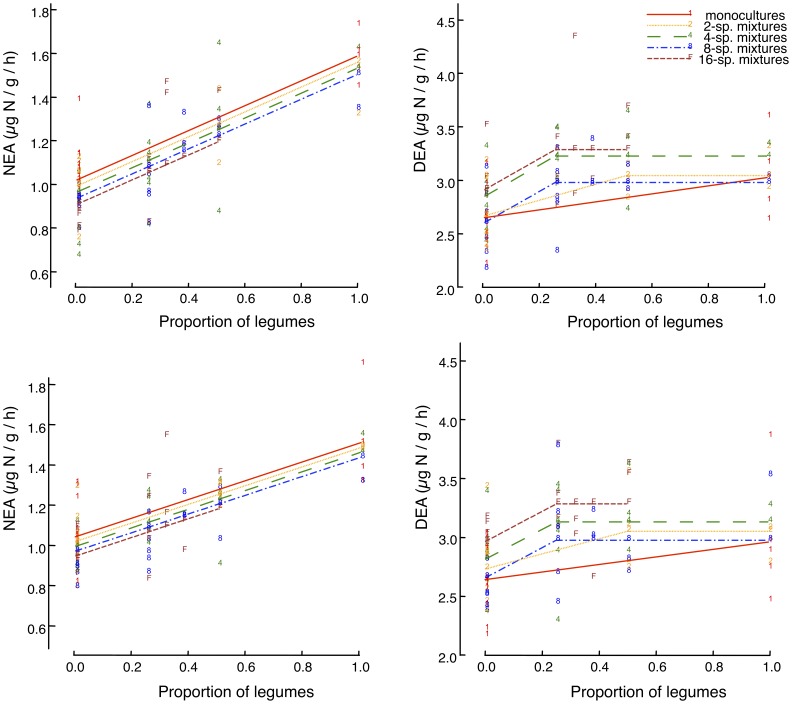
Unravelled effects of Legumes And Plant Species Richness On Soil Enzyme Activities. Changes in (left) NEA and (right) DEA as a function of the proportion of legumes within the plant community (x-axis) and plant species richness in the community (numbers associated to points). Data are presented for (top) 2006, and (bottom) 2007. The lines connect fitted values from corresponding multiple regression models with significance levels presented in Tables 1 and S1. For example, for the NEA-2006 plot (top, left), the increase of NEA with the proportion of legumes at a given richness level is significant at p<0.001 and the continuous increase of NEA with richness is significant at p = 0.009. Note that for NEA the five regression lines are not all significantly different from each other, but reflect the significant change with log richness (for each doubling of species richness the lines move down by a constant distance), which is different for DEA.

NEA increased with the percentage of legumes in a community, but decreased with species richness, the latter effect size being smaller (Fig. 1 and 2, Table 1). On average, NEA increased by about 60% from communities without legumes to communities containing only legumes. DEA increased in the presence of legumes and with plant species richness, but decreased with the percentage of grasses (Fig. 1 and 2, Table 1). A small proportion of legumes was sufficient to notably increase DEA, while increasing the proportion of legumes beyond 25% had no further effect (Fig. 2). DEA was on average 20% higher at highest plant species richness than in monocultures. Multiple regression analysis revealed that the different components of plant diversity explained 60% and 44% of the overall variation in NEA and DEA, respectively (after having accounted for spatial variation). Functional group composition explained 13.4 and 1.6 times more variation of NEA and DEA, respectively, than plant species richness alone (Fig. 1).

We verified that these results remained valid when including all the monocultures (i.e. the 16 monocultures on large plots plus the 44 additional ones on small plots) in the multiple regression analysis (not shown). Furthermore, NEA and DEA were very similar between the two study years (NEA_2007_ = 0.988 * NEA_2006_, r^2^ = 0.70, n = 88; DEA_2007_ = 0.953 * DEA_2006_, r^2^ = 0.69, n = 88), and the relative effects of the different plant diversity components were also very similar (Table S1).

### Relationships between Soil Enzyme Activities and Abundance of (de)nitrifiers

**Figure 3:**
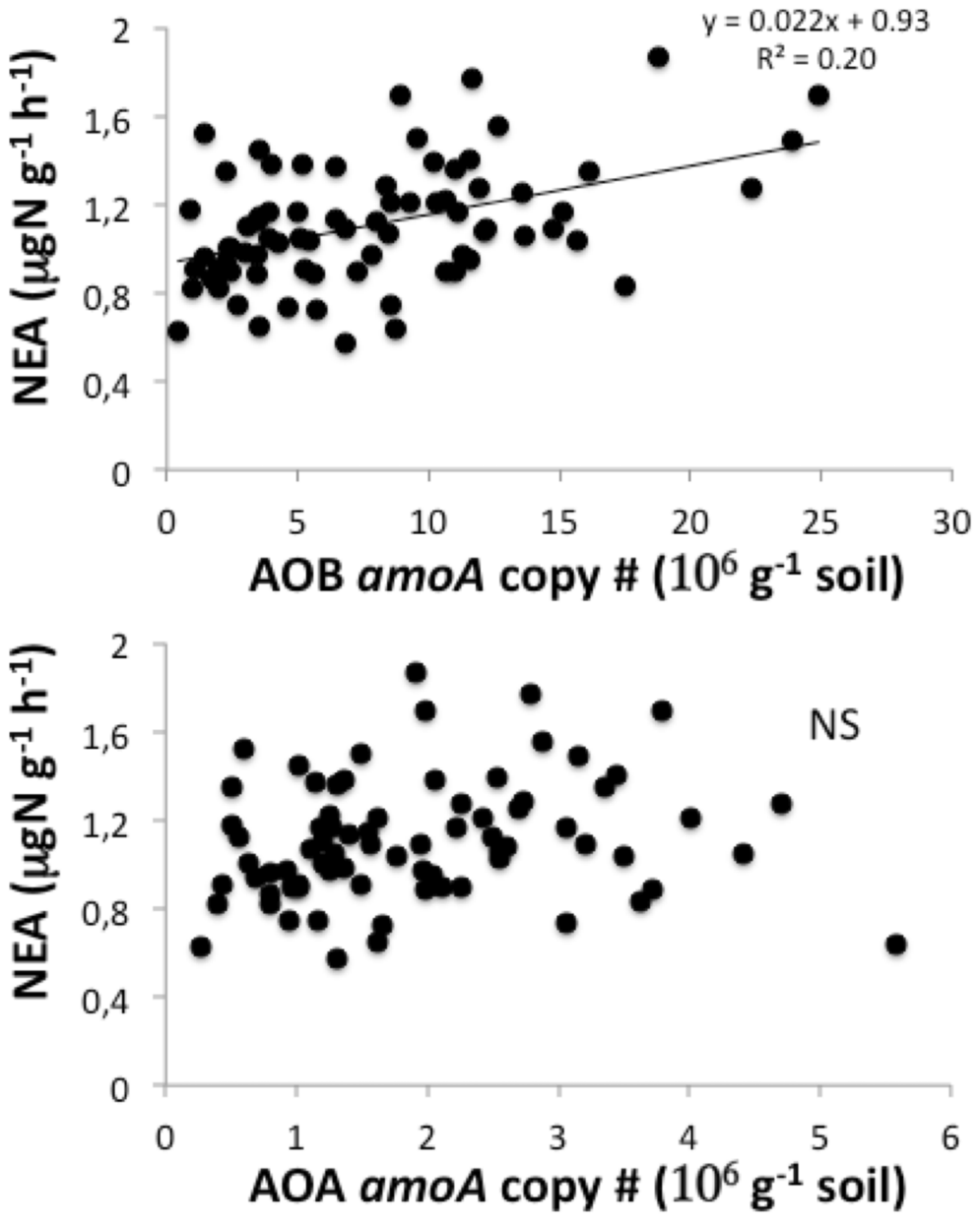
Relationship Between Plant Diversity-induced Changes In Nitrifier Abundance And Nitrifying Enzyme Activity. Correlations between NEA and (top) the abundance of ammonia oxidizing bacteria assessed by the number of their *amoA* sequences, and (bottom) the abundance of ammonia oxidizing archaea assessed by the number of their *amoA* sequences, in 2006. When significant (p<0.05), the linear regression is drawn.

The abundance of ammonia oxidizing bacteria, AOB, was five-fold higher than that of ammonia oxidizing archaea, AOA, in Jena soils (Fig. 3). A significant, although weak, correlation was observed between NEA and the AOB abundance: on average, soils with high nitrifying potential had significantly higher copy number of AOB *amoA* sequences (Fig. 3 top). Although the copy number of *amoA* sequences from AOA was significantly correlated to that of AOB (AOAnumber = 0.21*AOBnumber; r^2^ = 0.25), no significant relationship was observed between NEA and AOA abundance (Fig. 3 bottom).

**Figure 4:**
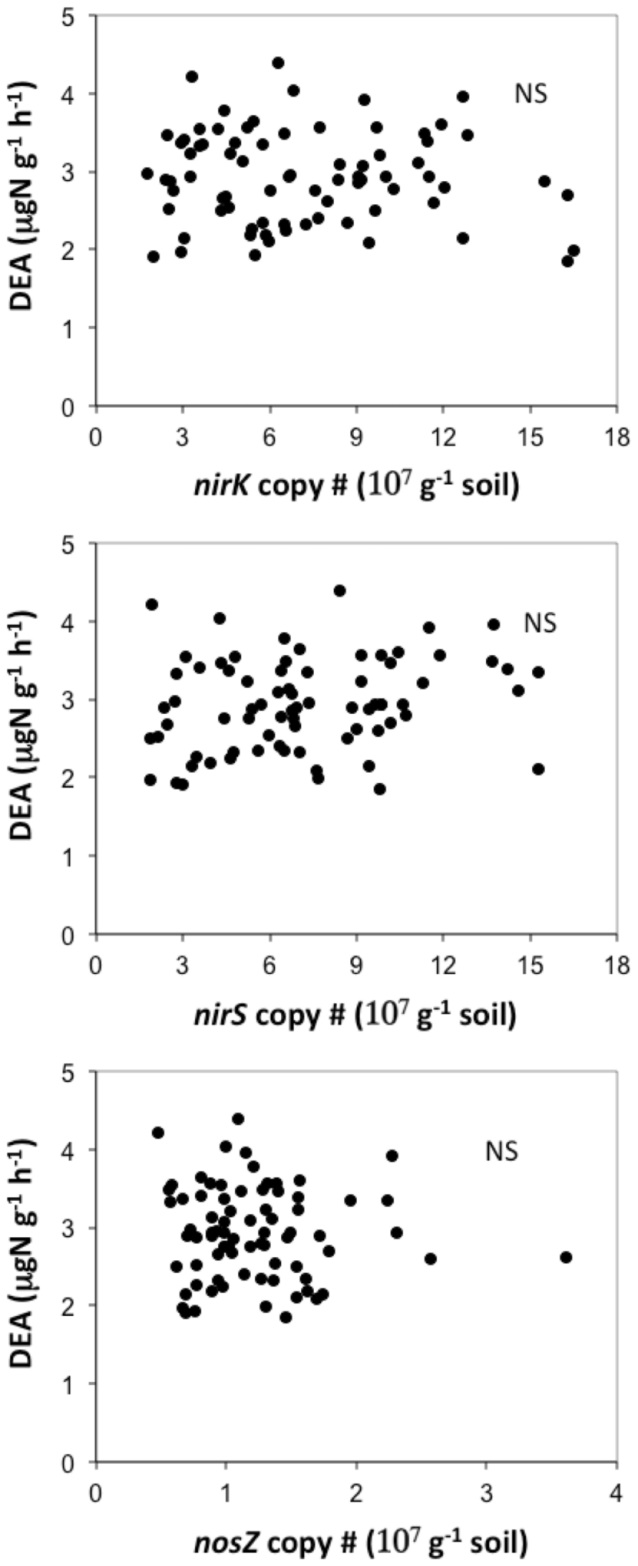
Relationship Between Plant Diversity-induced Changes In Denitrifier Abundance And Denitrifying Enzyme Activity. Correlations between DEA and the abundance of (top) *nirS*–harbouring denitrifiers, (middle) *nirK*–harbouring denitrifiers and (bottom) *nosZ*–harbouring denitrifiers in soil, in 2006. No significant correlation was observed (all p>0.05).

No significant correlation was observed between DEA and the abundance of denitrifiers (Fig. 4). This result was obtained for whatever targeted sequences we used to assess denitrifier abundance, i.e. for *nirS-, nirK-* or *nosZ-*harbouring denitrifiers. The abundance of the three sequence types were correlated with each other (*nirK* sequence number = 0.92*nirS sequence number, r^2^ = 0.30; and *nirS* sequence number = 5.66*nosZ sequence number, r^2^ = 0.26).

### Role of Environmental Variables and Microbial Abundances for the Relationships between Components of Plant Diversity and Soil Enzyme Activities

Significant correlations were observed between each soil enzyme activity and some environmental variables. In particular, DEA was significantly correlated to both soil nitrate concentration (p<0.001, Figure S2) and soil moisture (p<0.001, not shown), whereas no significant relationship was observed with DOC. Similarly, NEA was significantly correlated (p = 0.009) to soil ammonium concentration (not shown).

**Figure 5:**
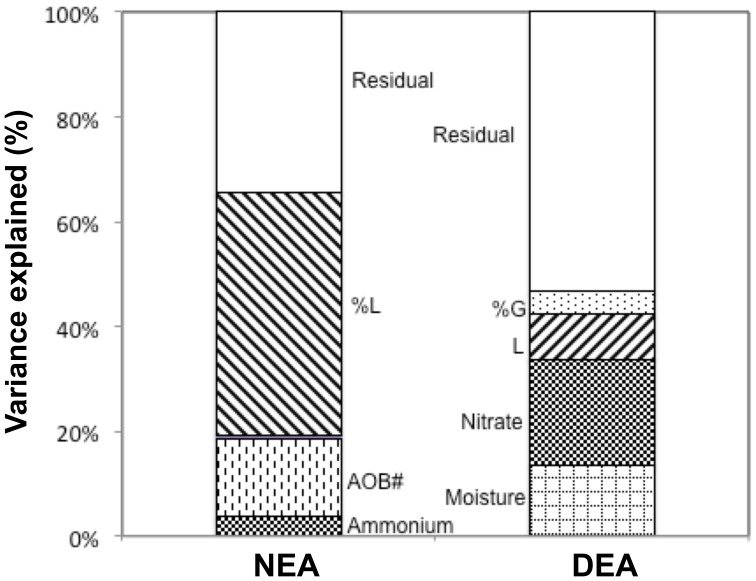
Soil Conditions And Microbial Abundances Partly Explaining Plant Diversity Effect On Soil Enzyme Activities. Percentages of variation in nitrifying and denitrifying enzyme activities, respectively NEA and DEA, significantly explained by environmental soil variables and microbial abundance, and remaining percentages of variation significantly explained by components of plant diversity (2006 data). See Fig. 2 for legend, and table 2 for full regression analyses and significance levels.

Including plant diversity components, soil environmental variables, and microbial abundances in our multiple regression analyses, we found that a substantial part of the plant diversity effect on NEA and DEA was attributable to plant-induced changes in key environmental variables and microbial abundances (Table 2, Fig. 5). In particular, all the effect of plant species richness on NEA was explained by an effect on the abundance of ammonia oxidizers: the richness effect was not significant anymore when AOB abundance was first accounted for. Furthermore, one fifth of the legume effect on NEA was explained by plant-induced variations in soil ammonium concentration. However, nitrifier abundance and soil ammonium explained only 19% of the variation in NEA, whereas an additional 46% of the variation was related to the percentage of legumes without being explained by any measured covariable.

All the effect of plant species richness on DEA was explained by an effect on soil moisture: the species richness effect was not significant anymore when moisture was first accounted for (Table 2, Fig. 5). Furthermore, plant diversity-induced changes in soil moisture and nitrate concentration explained 33.1% of the variation in DEA values, i.e. 76% of the observed effects of the different plant diversity components on DEA, whereas dissolved organic carbon was not identified as a key driver for DEA. Only 12.6% of the variation in DEA was related to the presence of legumes and the percentage of grasses without being explained by a measured soil environmental variable (Fig. 5).

**Figure 6:**
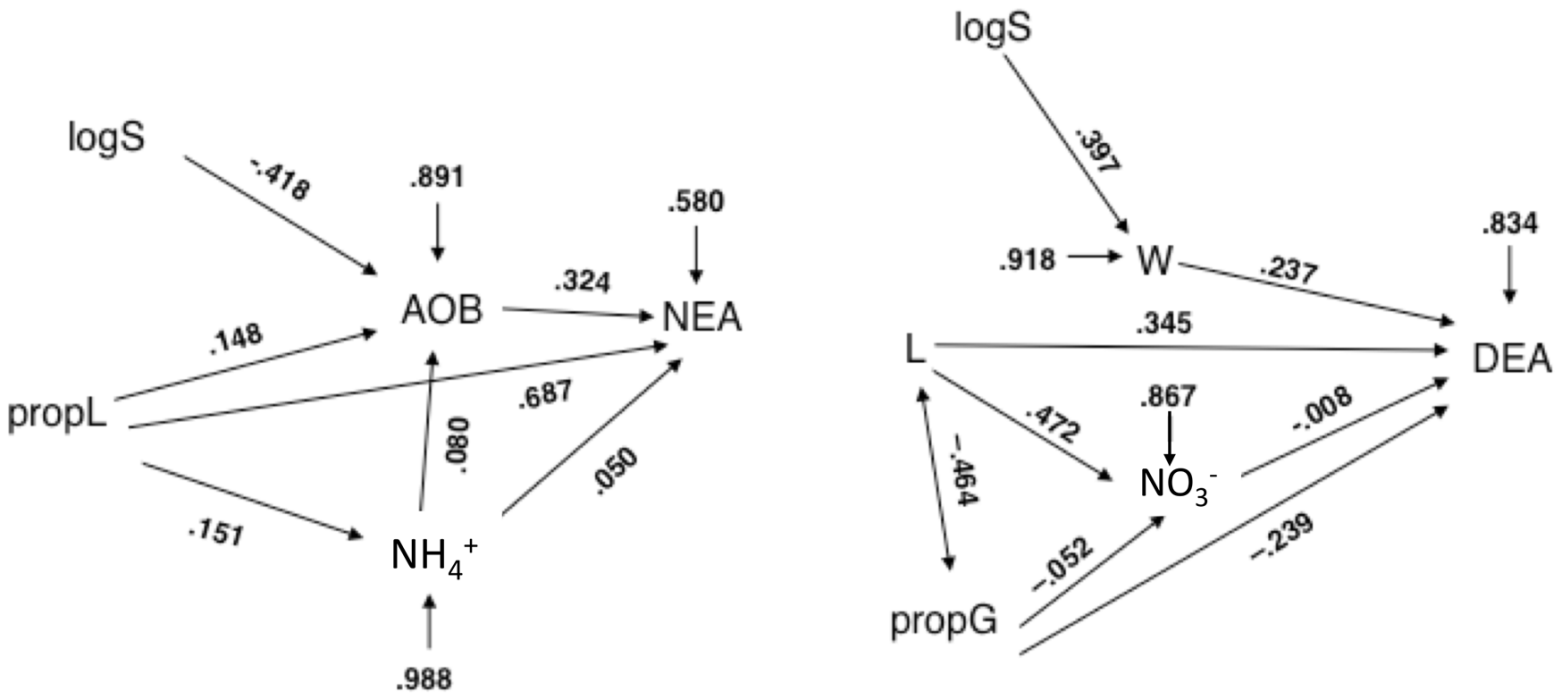
Path Diagram: Plant Diversity Components, Environmental Soil Variables, Microbial Abundances and Enzyme Activities. Path diagram for effects of plant diversity components on NEA and DEA, showing how observed effects can be explained entirely or partly by indirect effects via microbial abundance and key environmental drivers for (right) nitrifiers and (left) denitrifiers (2006 data). Lack of fit of path models was not significant for NEA (χ2 = 1.38, d.f. = 3, P = 0.71) or significant for DEA (χ2 = 19.53, d.f. = 7, P = 0074). Path coefficients indicate by how many standard deviations the effect variable would change if the causal variable were changed by one standard deviation.

Path analyses were used to provide a comprehensive analysis of predicted cause-effects relationships between plant diversity components, microbial abundance and soil environmental variables, and NEA or DEA (Fig. 6). Our results confirmed that all the negative effect of plant species richness on NEA was mediated by a decrease in the abundance of ammonia oxidizers. The positive effect of the percentage of legumes on NEA was partly explained by a positive effect on the abundance of ammonia oxidizers, and to a lesser extent on ammonium concentration (Fig. 6).

Similarly, path analysis showed that the positive effect of plant species richness on DEA was entirely mediated by an increase in soil moisture (Fig. 6). Both the positive effect of the presence of legumes and the negative effect of the proportion of grasses on DEA were partly explained by respectively a positive and a negative effect on nitrate concentration. However, apparent direct effects of the presence of legumes and the proportion of grasses on DEA remained significant (Fig. 6).

## Discussion

### Plant Diversity Effect on Soil Enzyme Activities: a Matter of Microbial Abundance or Specific Activity?

In this study, a significant correlation was observed between NEA and the abundance of nitrifiers, in particular ammonia oxidizing bacteria. The observed relationship between NEA and AOB abundance is consistent with the correlations obtained between nitrification levels and the abundance of AOB in different ecosystems, e.g. [32], [36], [39]; but see [40]. The data published so far have shown a higher abundance of AOA than AOB in a range of soils [35], with indications that the AOA:AOB ratio increases with increased nutrient limitation. Lamb et al. [15] reported that the richness of non-legume plant species increased the prevalence of AOA relative to AOB. In contrast, we found here that AOB abundance was 5-fold higher than AOA abundance in the Jena fertile grassland soils, though the abundance of AOB and AOA were correlated. This is consistent with the report that AOB and AOA abundances were correlated in another fertile grassland system [36]. Given the higher number of AOB as compared to AOA in Jena soils, and given that transformation rates for ammonium into nitrite by AOA are currently assumed to be lower than for AOB [41], it can be assumed that the major part of nitrification in the grassland soils studied was related to AOB activity. Our results actually show that plant diversity conditioned NEA at least partly through the build-up of nitrifying bacteria.

In contrast, no correlation was observed between DEA and the abundance of denitrifiers, although three different, relevant genes were targeted to assess denitrifier abundance. Lack of or weak correlation between DEA and denitrifier abundance has often been reported for a range soil systems, e.g. [42], though significant correlations were sometimes reported [43]. This shows that plant diversity conditioned DEA not through changes in denitrifier abundance, but by influencing the specific activity of denitrifiers, i.e. the mean activity per denitrifying cell.

The contrasting results obtained for the two activities can be explained by the roles of those activities for the two types of microoganisms. Indeed, nitrification is the key activity through which ammonia oxidizing organisms acquire energy and grow, so that a significant coupling between changes in the activity and abundance of ammonia oxidizers is expected. In contrast, denitrification is a facultative activity for denitrifying organisms that switch to another heterotrophic metabolism when oxygen availability is sufficient. Thus the enzyme pool per denitrifying cell can be strongly affected by plant diversity-induced changes in environmental conditions in soil. This shows that different representations are needed to model the relationships between plant diversity and soil functions: for nitrification, nitrifier abundance is a key variable affected by plant diversity-induced changes in soil conditions, whereas denitrifier specific activity is the key variable affected for denitrification.

### Underlying Drivers of the Effects of Plant Diversity Components on Enzyme Activities

The results of the multiple regression and path analyses show that the presence or proportion of legumes, the proportion of grasses along with plant species richness were key determinants of NEA and DEA, legumes playing a major role for NEA. Several studies have already reported the key role of legumes on ecosystem processes, via an associated enhancement of soil N availability, in biodiversity experiments [22], [23], [37], [44], [45], though climatic and soil nutritional limitations can dampen legume effects [9], [21], [46]. In particular, an effect of legumes was reported on nitrification, denitrification and nitrous oxide fluxes [24] and nitrifiers [14]. In addition, DEA was negatively related to the proportion of grasses in a plant community. This is consistent with previous reports showing that a legume-induced increase in soil mineral N can be compensated by the ability of grasses to effectively take up mineral N even at very low concentrations in the soil due to their extensive rooting system [23], [37], [44], [47]. However, multiple regression and path analyses show that increased ammonium and nitrate concentrations in soil only partly explained the legumes effects on NEA and DEA, respectively. Similarly, decreased soil nitrate concentration only partly explained the grasses effects on DEA. Actually soil mineral N concentrations under field conditions result from various N processes depleting or increasing the mineral N pool; snapshot measurements of mineral N concentrations reflect quasi-steady state concentrations rather than turnover and thus are questionable proxies of actual N availability [48]. For example, Malchair et al. [14] reported that soil ammonium concentration does not reflect ammonium availability to nitrifiers, which could explain the lack of relationship between NEA or nitrifier abundance and ammonium concentration. This would explain why the residual direct effect of legumes or grasses on NEA or DEA is still significant even after accounting for an indirect effect through ammonium or nitrate concentration (see figure 5). Interestingly, our results show that legumes increased DEA regardless of their abundance, likely due to a change in the factor(s) limiting the specific activity of denitrifers (i.e. activity per denitrifying cell). The limiting factor would be N availability for non-legume plant mixtures, but 25% of legumes in a community (Figure 2) would be enough to obtain non limiting nitrate concentrations; C or anaerobic microsite availability would thus become limiting for denitrifiers in plant mixtures with at least 25% of legumes.

Our results also show a positive effect of plant species richness sensu stricto on DEA consistently with a few other studies [49]. We show that this effect was entirely mediated by increased soil moisture. This is consistent with a recent study showing that plant species richness effects on in-situ ammonification rates were mediated through soil moisture [50]. For a Mediterranean grassland it has been reported that soil moisture explained 60% of the variation in DEA across a full factorial combination of simulated components of global change [51]. Here, we show that this also applies across a gradient of plant species richness, though soil nitrate had also an important role. In contrast, NEA was negatively related to plant species richness: this was entirely explained by a negative effect of richness on the abundance of ammonia oxidizers, which could be partly due to the richness-induced increase in soil moisture and/or decrease in soil ammonium availability, but this could not be captured in our path analysis.

Though plant diversity effects cannot be generalised without considering the level of soil resources [52], the experimental setup of our study allowed us to efficiently disentangle the respective effects of plant species richness and plant functional group composition (the presence and relative abundance of each functional group in the plant community) on NEA and DEA. The interaction between legume effect and effects of other plant diversity components was not significant: a positive effect of legume relative abundance occurred independently of species richness, and the effects of species richness occurred independently of legume abundance. Moreover, our results show that these plant diversity components conditioned NEA at least partly through the build-up of nitrifying bacteria, likely driven by changes in soil ammonium availability. In contrast, plant diversity components conditioned DEA mainly through changes in soil moisture and nitrate that influenced the specific activity of denitrifiers rather than their abundance.

### Accounting for Temporal and Spatial Variability of NEA and DEA

Nitrogen dynamics and availability in the soil are governed by many interacting processes such as ammonification, nitrification, uptake by plants and microorganisms, denitrification, leaching and volatilization [53]. These processes can be highly variable in time and space [54], [55]. To evaluate our accounting for spatial variability of soil enzyme activities in the present study, we compared - for 28 plots randomly selected - the mean of activity values obtained in the 10 individual soil cores sampled per plot to the value obtained in the composite soil sample derived from the same plot during the 2006 field campaign. Strong correlations were observed between the two for both DEA (DEA_composite_ = 1.06 * DEA_mean_, r^2^ = 0.92, n = 28 plots) and NEA (NEA_composite_ = 1.00 * NEA_mean_, r^2^ = 0.79, n = 28 plots), thus demonstrating that our composite soil samples adequately integrated within-plot spatial variability in soil activities.

Temporal variability in nitrification and denitrification can be large: for instance, large emission bursts of nitrous oxide from soil in the field are triggered within hours of wetting events, making them difficult to integrate [55]. However, NEA and DEA are related to nitrification and denitrification by integrating the activity of living nitrifiers and denitrifiers over periods in the order of at least a few days in the field. Moreover, the observed patterns were consistent over time: (i) activities measured in both years were strongly correlated, and (ii) the drivers identified were the same in both years. Additional evidence for the constancy of these drivers is indicated by the consistency over seasons and years of both the soil moisture patterns and the effects of legumes and grasses on soil N concentrations in Jena plots [37]. Overall, these results support the robustness of our findings.

### Conclusion

The main objective of biodiversity experiments with artificially established model ecosystems such as the Jena experiment is to shed light on the driving mechanisms of biodiversity–ecosystem functioning relationships [56]. Here, we identified and ranked the key components of plant diversity and associated mechanisms that drive enzyme activities associated to two belowground processes (nitrification and denitrification), which is rarely quantified in biodiversity–ecosystem functioning studies. Legume effect and effect of plant species richness per se represented 93% and 7% of total plant diversity effects on NEA, respectively. In contrast, the effects of legumes, grasses and plant species richness per se represented 50%, 11% and 39% of total plant diversity effects on DEA, respectively. Our results highlight the main biological processes explaining those patterns: plant diversity components conditioned NEA largely through the build-up of nitrifying bacteria likely responsive to soil ammonium availability. In contrast, plant diversity components conditioned DEA mainly through changes in the specific activity of denitrifiers that was influenced by changes in soil moisture and nitrate, the latter explaining 76% of the total plant diversity effects observed on DEA.

Our results demonstrate that the response of soil microbial enzyme activities to plant diversity varies qualitatively according to the type of activity, which should be well represented in ecosystem models. For non-facultative functions like nitrification, accounting for the changes in the abundance of microbial groups is a major step forward in understanding and predicting changes in soil functioning, which are expected to be relatively long lasting since significant changes in nitrifier abundance in such grasslands typically operate over period of months under moderate disturbance regime [36]. For facultative functions like denitrification, plant diversity-induced changes in the microbial activity depends mainly on changes in the specific activity of microorganisms modulated by changed soil conditions (here oxygen and to a lesser extent nitrate availability), which is likely more dynamic and, e.g., strongly weather-dependent. Ecosystem models should take this into account to adequately represent aboveground/belowground couplings induced by changes in plant diversity.

## Supporting Information

Figure S1 Designs used to manipulate different components of plant diversity. The figure compares the design of the Jena Experiment used to manipulate different components of plant diversity to typical designs used in previous grassland plant species assemblage experiments aiming at studying biodiversity-ecosystem functioning relationships.
(DOC)

Figure S2 Relationship between denitrifying enzyme activity and nitrate concentration in soil for October 2006.
(DOC)

Table S1 ANOVA results of the multiple regression models fitted for the two dependent variables nitrifying and denitrifying enzyme activities for October 2007, using plant biodiversity components only as explanatory variables.
(DOC)
