# Supplementary material for: Soil Environmental Conditions and Microbial Build-Up Mediate the Effect of Plant Diversity on Soil Nitrifying and Denitrifying Enzyme Activities in Temperate Grasslands
Source: PLoS One. 2013 Apr 17;8(4):e61069. doi: 10.1371/journal.pone.0061069 (PMC3629084; doi:10.1371/journal.pone.0061069)

**Appendices for the manuscript “*Soil environmental conditions and buildup of microbial communities mediate the effect of plant diversity on nitrifying and denitrifying enzyme activities in temperate grasslands*” by Le Roux X. et al. (*PLOS One*, 2013)**

**Figure S1.** Designs Used to Manipulate Different Components of Plant Diversity

Fraction of grassland plots containing legumes as a function of sown plant species richness in (Left) a design typical of most grassland plant species assemblage experiments aiming at studying biodiversity-ecosystem functioning relationships, here the BIODEPTH experiment, and (Right) the Jena Experiment. Disc area is proportional to the number of plots. The pattern is similar for other functional groups (not shown).


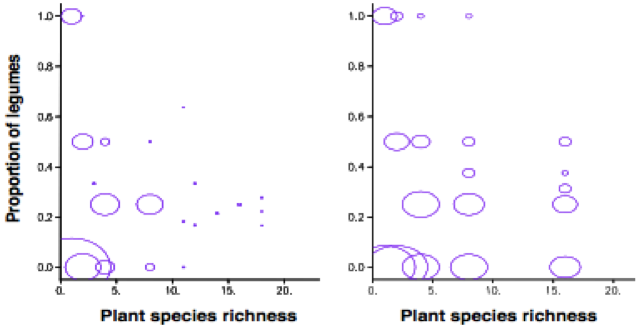

Supplement: Figure S1 — Designs used to manipulate different components of plant diversity. The figure compares the design of the Jena Experiment used to manipulate different components of plant diversity to typical designs used in previous grassland plant species assemblage experiments aiming at studying biodiversity-ecosystem functioning relationships. (DOC) [file pone.0061069.s001.doc]
