# Supplementary material for: Soil Environmental Conditions and Microbial Build-Up Mediate the Effect of Plant Diversity on Soil Nitrifying and Denitrifying Enzyme Activities in Temperate Grasslands
Source: PLoS One. 2013 Apr 17;8(4):e61069. doi: 10.1371/journal.pone.0061069 (PMC3629084; doi:10.1371/journal.pone.0061069)

**Appendices for the manuscript “*Soil environmental conditions and buildup of microbial communities mediate the effect of plant diversity on nitrifying and denitrifying enzyme activities in temperate grasslands*” by Le Roux X. et al. (*PLOS One*, 2013)**

**Figure S3.** Relationship between denitrifying enzyme activity and nitrate concentration in soil. Data are for October 2006. The regression line (p<0.001) is indicated.


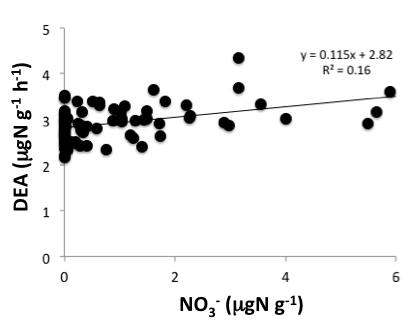

Supplement: Figure S2 — Relationship between denitrifying enzyme activity and nitrate concentration in soil for October 2006. (DOC) [file pone.0061069.s002.doc]
