# Supplementary material for: Soil Environmental Conditions and Microbial Build-Up Mediate the Effect of Plant Diversity on Soil Nitrifying and Denitrifying Enzyme Activities in Temperate Grasslands
Source: PLoS One. 2013 Apr 17;8(4):e61069. doi: 10.1371/journal.pone.0061069 (PMC3629084; doi:10.1371/journal.pone.0061069)
Supplement: Table S1 — ANOVA results of the multiple regression models fitted for the two dependent variables nitrifying and denitrifying enzyme activities for October 2007, using plant biodiversity components only as explanatory variables. (DOC) [file pone.0061069.s003.doc]

**Appendices for the manuscript “*Soil environmental conditions and buildup of microbial communities mediate the effect of plant diversity on nitrifying and denitrifying enzyme activities in temperate grasslands*” by Le Roux X. et al. (*PLOS One*, 2013)**

**Table S2.** ANOVA results of the multiple regression models fitted for the two dependent variables nitrifying and denitrifying enzyme activities for October 2007, using plant biodiversity components only as explanatory variables. LogSR and SR respectively refer to the decomposition of the species richness factor into a contrast for log-linear richness and remainder (deviation from log-linearity). Values are presented only for variables that had a significant effect on activity. Abbreviations are as in Table 1.

October 2007:

| **Nitrifying activity** | |  |  |  |
| --- | --- | --- | --- | --- |
| Source of variation | d.f. | s.s. | m.s. | F pr. |
| Spatial variation | 4 | 2.5621 | 0.64052 | <.001 |
| LogSR | 1 | 0.1259 | 0.12593 | 0.021 |
| Legume abundance (% sown) | 1 | 1.6488 | 1.64882 | <.001 |
| Residual | 68 | 1.5397 | 0.02264 |  |
| Total | 74 | 5.8765 | 0.07941 |  |

| **Denitrifying activity** | | |  |  |
| --- | --- | --- | --- | --- |
| Source of variation | d.f. | s.s. | m.s. | F pr. |
| Spatial variation | 6 | 19.8836 | 3.3139 | <.001 |
| logSR | 1 | 1.1233 | 1.1233 | 0.002 |
| SR | 3 | 0.5893 | 0.1964 | 0.159 |
| Presence of legumes | 1 | 1.7832 | 1.7832 | <.001 |
| Grass abundance (% sown) | 1 | 0.8101 | 0.8101 | 0.009 |
| Residual | 62 | 6.8199 | 0.1100 |  |
| Total | 74 | 31.0093 | 0.4190 |  |
